# Food Insecurity Is Highly Prevalent Amongst Patients With Chronic Liver Disease in Northern England: A Cross‐Sectional Analysis

**DOI:** 10.1111/jhn.70173

**Published:** 2025-11-27

**Authors:** Rachel Howarth, Thomas Crame, Stuart McPherson, Quentin M. Anstee, Laura Haigh

**Affiliations:** ^1^ Newcastle NIHR Biomedical Research Centre Newcastle upon Tyne Hospitals, NHS Trust Newcastle upon Tyne UK; ^2^ Translational and Clinical Research Institute, Faculty of Medical Sciences Newcastle University Newcastle upon Tyne UK

**Keywords:** alcohol‐related liver disease, food insecurity, health inequalities, metabolic dysfunction associated liver disease

## Abstract

**Introduction:**

Liver disease disproportionately affects the most economically disadvantaged. Since 2019, Food insecurity (FI) prevalence has increased by 26% in Europe and 214% in the UK. FI compromises nutritional status and may also hamper the adoption of recommended dietary lifestyle advice. We aimed to assess the extent of FI and its associated factors among patients with liver disease.

**Methods:**

This cross‐sectional study recruited consecutive patients attending hepatology outpatient services at a tertiary centre between October 2023 and March 2024. Food security status was assessed using validated tools: the United States Department of Agriculture's Six‐item Household Food Security Survey and the Hunger Vital Signs Two‐Item Screener. Logistic regression was performed to assess associations between FI and clinico‐sociodemographic variables.

**Results:**

103 (24%) of 426 patients screened were identified as food insecure. Predictive variables for FI included: high risk/dependent alcohol intake (OR 2.7, 95%CI 1.1–6.8, *p* = 0.03), alcohol related liver disease (ArLD) (OR 1.9, 1.1–3.1, *p* = 0.02), cirrhosis (OR 1.6, 1.1–2.6, *p* = 0.03) and depression (OR 1.7, 1.1–2.8, *p* = 0.023). After adjustment for co‐morbidities, clinical and socio‐demographic characteristics, ArLD (AOR = 3.2, 1.3–8.0, *p* = 0.013), metabolic dysfunction associated steatotic liver disease (MASLD) (AOR = 2.3, 1.1–5.0, *p* = 0.03) and cirrhosis (AOR = 1.9, 1.0–3.0, *p* = 0.034) were found to be associated with FI.

**Conclusion:**

FI was higher amongst patients with liver disease than the background population, and associated with ArLD, MASLD and cirrhosis. These results highlight an important paradox wherein FI contributes to obesity, so promoting consequent end‐organ damage in MASLD, and compromises nutritional status. It is also a barrier to implementation of recommended diet lifestyle advice. Healthcare professionals should consider FI within holistic patient assessment.

## Summary

- Food insecurity prevalence is significantly higher amongst patients with liver disease compared to the general population. It is particularly associated with ArLD, MASLD and presence of cirrhosis.
- Food insecurity presents a barrier to implementation of diet and lifestyle advice, with potential implications in disease management, physical and mental wellbeing.
- Our findings highlight a need for routine consideration of food security status amongst hepatology patients.

## 1 Introduction

Liver disease is the third leading cause of death in the United Kingdom (UK), with mortality rates having increased by 400% since 1970 [1]. This rising burden parallels the increase in excess alcohol consumption, obesity, and viral hepatitis [1]. Liver disorders disproportionately affect the most economically disadvantaged, exacerbating health inequalities [2]. Premature deaths from liver disease are four times higher in the most deprived areas of the UK, compared to the most affluent [2]. The majority of liver disease is preventable. To address lifestyle risk factors of liver disease, promoting healthy lifestyles, and dietary patterns may be recommended [1, 3, 4]. However, there are important evidence gaps that limit the delivery of effective lifestyle advice in clinical practice.

Food insecurity is defined as the inability of individuals and households to obtain an adequate and nutritious diet in socially acceptable ways, or the uncertainty that they will be able to do so [5, 6]. Root causes of food insecurity are predominantly economic, with financial constraint being the primary cause amongst UK households [7]. It exists on a spectrum from mild, where there is doubt about obtaining food, to severe, where individuals and households may go without food for full days at a time [5]. In the UK, food insecurity prevalence has increased by 214% since 2020 based on a nationally representative population [8]. This is underpinned by a combination of factors, including increased fixed household costs and food prices, alongside a prolonged period of inflation‐adjusted pay and benefits cuts [9]. Food insecurity might affect the adoption of recommended lifestyle changes, with potential for increasing impact as prevalence rises. However, it remains uncertain if healthcare professionals routinely screen for and address this complex issue in clinical practice.

The poorest fifth of UK households would need to spend 50% of their disposable income on food to meet the cost of the Eatwell Guide, a government recommended model of healthy eating [10]. Further, food insecurity is associated with substitution of nutrient dense, low energy foods for cheaper, energy dense foods higher in sugar and saturated fat [11]. Emergent evidence indicates the existence of a paradox wherein food insecurity contributes to both obesity and compromised nutritional status, through energy‐dense but nutrient poor diets [11]. Moreover, food insecurity negatively impacts psychological wellbeing including sleep disorders, anxiety and depression [12]. Therefore, it is crucial for healthcare professionals to accurately identify and provide effective advice for food‐insecure individuals.

The existing evidence base concerning food insecurity primarily focuses on the general population [5, 13]. However, current evidence does not define the extent to which food insecurity affects people with liver disease or the associations between food insecurity, liver disease and related clinical and sociodemographic factors. The aim of this study was to assess the extent of food insecurity and its associations with chronic liver disease amongst patients attending outpatient hepatology services at a tertiary centre. Our objectives were firstly to measure the prevalence of food insecurity amongst patients with chronic liver disease. Secondly, to identify any relationships between food insecurity and clinical and sociodemographic factors in patients with chronic liver disease.

## 2 Materials and Methods

### 2.1 Study Subjects

The study was a cross‐sectional analysis of data collected from all eligible patients presenting consecutively to outpatient appointments at the tertiary liver unit in Newcastle‐upon‐Tyne Hospitals National Health Service (NHS) Foundation Trust, North‐East England. Patients were recruited from clinics serving adults, focused on general hepatology including a broad spectrum of aetiologies. The inclusion criteria were a diagnosis of liver disease and an age of 18 years or older. Patients were excluded if they failed to complete the food insecurity screening process, either due to non‐return of the screening tool or incomplete responses. Caldicott approval (ID: 10067) was gained for this study from Newcastle Upon Tyne Hospitals NHS Foundation Trust.

### 2.2 Food Insecurity Screening Process

Patients at consecutive clinics during the data collection period (10th October 2023 to 19th March 2024) were invited to complete the food insecurity screening process. The data collection period was selected to maximise the recruitment period, whilst ensuring optimal staff and participant engagement. Further, it allowed representation of seasonable variables over the Christmas period.

Food insecurity screening was a new aspect of clinical care in the outpatient service. Implementation of food insecurity screening was informed by Patient and Public Involvement and Engagement groups, and facilitated by an academic dietitian, who mentored clinic‐based dietitians. Informal training was provided to physicians and clinic‐based staff. Dietitians were present in clinic throughout the study, ensuring standardised practices.

Staff nurses and healthcare assistants offered patients the opportunity to complete the screening process upon initial presentation in clinic. An overview of the study, outline of the screening process and detailed information on data collection and handling was discussed with patients. Completion of the screening process constituted implied consent.

The screening process comprised the completion of two validated tools to determine food security status: (i) the United States Department of Agriculture (USDA)'s Six‐item Household Food Security Survey and (ii) the Hunger Vital Signs Two‐Item Screener [14, 15]. The questions contained in both the USDA and Hunger Vital Signs tools solely concern food insecurity arising from financial constraint [14, 15]. The USDA six‐item survey assesses the respondent's dietary adequacy and sufficiency over the preceding 12‐month period. Food insecurity was determined by affirmative responses totalling two or more, whilst scores of less than two are indicative of food security [14]. The Hunger Vital Signs screener measures a respondent's food access and anxiety during the past 12 months. One or more affirmative response was used to categorise food insecurity [15]. Participants were classified as food insecure where their responses to one or both measures indicated this. We opted to utilise both the USDA Food Security Survey Module, which provides detailed prevalence estimates comparable with national data [12], and the Hunger Vital Sign, a brief screener recommended for use in healthcare settings [13], due to their relevance for both epidemiologic and clinical circumstances. They were combined in one food insecurity screening process provided to study participants.

Once the tools were completed, these were scored by either doctors or dietitians present in clinic. Where food insecurity was identified, patients were offered an immediate brief ( ~ 10 min) intervention appointment with a dietitian. This focused on food insecurity screening and signposting and did not include clinical dietetic assessment or intervention. The dietetic intervention incorporated 12 additional questions to extend the USDA 6‐item survey already completed by patients to the USDA 18‐item Household Food Security Survey. This 18‐item tool further categorises food security status as ‘low food security’ and ‘very‐low food security’ (six or more affirmative responses in adult‐only households, eight or more affirmative responses in households with children) [12]. The brief intervention included nutritional counselling, written education in the form of the British Dietetic Association food fact sheet ‘Eat well, spend less’ [16] and signposting to telephone and internet contacts for support within civil society including local councils, charities, food banks and community pantries.

### 2.3 Clinical and Sociodemographic Variables

Liver disease aetiology, presence of cirrhosis, and relevant information on co‐morbidities were extracted from electronic patient records. Data was collected on co‐morbidities with known associations to liver disease: features of metabolic syndrome (type two diabetes mellitus, hypertension, and dyslipidaemia), cardiovascular disease, thyroid disorders, anxiety and depression, psoriasis and polycystic ovary syndrome (PCOS), body mass index (BMI), and haemoglobin A1c (HbA1c). Body mass index was categorised in 5–10 kg/m^2^ increments. HbA1c was classified based on generally accepted diagnostic ranges for diabetes: </= 42 mmol/mol (normal), 42–48 mmol (pre‐diabetes), 48–57 mmol/mol (diabetes), >/= 58 mmol/mol inadequately controlled diabetes) [17].

Participants' age, partial postcode, marital status, ethnicity, smoking status and alcohol intake were obtained from electronic records where available. Age was reported as ranges (18–24 years, then in decade increments up to 65 years and over). Partial postcode was used to identify local authority, with subsequent categorisation according to Index of Multiple Deprivation (IOD) Rank, which classifies small geographical areas in the UK based on multiple domains of deprivation. Localities are assigned a score and relative rank based on 7 domains, with 1 being the most deprived [18, 19]. Alcohol intake was classified by the 3‐item Alcohol Use Disorders Identification Test (AUDIT‐C), as the standard measure utilised in the service. Within the screening process, patients were asked to indicate the primary retail outlet predominantly used for their food shopping. Multiple responses were typically given to this question, which were collated as frequency data based on retailer price point (lower, mid and higher cost) for an average household shop in March 2024 [20].

### 2.4 Statistical Analysis

Participant responses to the food insecurity screening tool, clinical and sociodemographic data were pseudonymised and compiled on Microsoft Excel® version 2016. Descriptive statistics were performed to summarise sample characteristics and reported as sample size (*n*) with percentage and mean ± standard deviation (SD).

A one‐sample proportion test was used to assess the null hypothesis that food insecurity prevalence amongst our sample would not differ from the background population. Shapro‐wilk and Q‐Q plot normality tests were conducted as appropriate, with non‐normal distribution of data found throughout. To assess differences between food secure and food insecure groups, Chi square, or Fisher‐Freeman‐Hamilton tests (cell counts less than five), were performed for categorical data. Mann‐Whitney U tests were used for continuous data. Statistical significance was set at *p* < 0.05.

Unadjusted binary logistic regression analyses were performed to identify odds ratios for associations between food security status (food secure vs food insecure) and clinical and sociodemographic variables, where at least 10 events per variable existed. Missing data were excluded for valid food secure and insecure group percentages within variables. The testing protocol was repeated for low and very low food secure groups amongst those who had completed the dietetic‐led extended screening. Adjusted logistic regression was performed to explore relationships between food insecurity (based on the binary initial screening tool), liver‐disease aetiology and presence of cirrhosis, accounting for variables with differences between groups of *p* < 0.05.

All data analyses were generated using IBM SPSS software, version 29.

## 3 Results

### 3.1 Sample Characteristics

In total, 426 participants completed the screening process, representing a response rate of 67% (from 636 total clinic attendees). Nine screening tools were duplicates and therefore excluded from the analyses. The characteristics of the patients are shown in Table 1. Data for those who chose not to participate were not collected, as consent was not obtained.

**Table 1:** Sample socio‐demographic and clinical characteristics by food security status, based on the binary food insecurity screening tool comprising the USDA and Hunger‐Vital signs measures.

		Total	Food secure	Food insecure	*p* value
		*n* = 426	*n* = 323	*n* = 103	
Characteristics	Category	*n* (%)	*n* (%)	*n* (%)	
Age, years	18–24	7 (1.6)	5 (71.4)	2 (28.6)	< 0.001
	25–34	20 (4.7)	14 (70.0)	6 (30.0)	
	35–44	52 (12.2)	29 (55.7)	23 (44.2)	
	45–54	62 (14.6)	38 (61.3)	24 (38.7)	
	55–64	124 (29.1)	100 (80.6)	24 (19.4)	
	65 and over	161 (37.8)	137 (85.1)	24 (14.9)	
Gender	Male	237 (55.6)	177 (74.7)	60 (25.3)	0.539
Ethnicity	White British/other	400 (93.9)	303 (75.8)	97 (24.2)	0.399
	Black ‐ any black background	2 (0.5)	2 (100.0)	0 (0.0)	
	Asian ‐ any asian background	7 (1.6)	6 (85.7)	1 (14.3)	
	Other	3 (0.7)	1 (33.3)	2 (66.7)	
	Not stated	14 (3.3)	11 (75.6)	3 (21.4)	
Marital status	Married/civil partner	165 (60.4)	134 (81.2)	31 (18.8)	0.028
	Single	85 (31.1)	66 (77.6)	19 (22.4)	
	Divorced/separated/widowed	23 (8.4)	13 (56.5)	10 (43.5)	
Smoking status	Smoker	51 (12.0)	36 (70.6)	15 (29.4)	0.378
	Ex‐smoker	169 (39.7)	127 (75.1)	42 (24.9)	
	Nonsmoker	171 (40.1)	136 (79.5)	35 (20.5)	
Alcohol intake (Audit‐C)	0–4 (low risk)	361 (84.7)	274 (75.9)	87 (24.1)	0.006
	5–7 (increasing risk)	32 (7.5)	28 (87.5)	4 (12.5)	
	8–12 (high risk/dependence)	20 (4.7)	11 (55.0)	9 (45.0)	
BMI	18.5–24.9	85 (20.0)	63 (74.1)	22 (25.9)	0.287
	25–29.9	117 (27.5)	94 (80.3)	23 (19.7)	
	30–34.9	113 (26.5)	85 (75.2)	28 (24.8)	
	35–39.9	67 (15.7)	48 (71.6)	19 (28.4)	
	40–49.9	37 (8.7)	27 (72.9)	10 (27.0)	
	>/= 50	7 (1.6)	6 (85.7)	1 (14.3)	
HbA1c	< 42	195 (45.8)	152 (77.9)	43 (22.1)	0.513
	42–47	107 (25.1)	78 (72.9)	29 (27.1)	
	48–57	28 (6.6)	25 (89.3)	3 (10.7)	
	>/= 58	77 (18.1)	51 (66.2)	25 (32.5)	
Diagnosis	ArLD	82 (19.2)	54 (65.9)	28 (34.1)	0.002
	Viral hepatitis	51 (12.0)	40 (82.4)	11 (21.6)	
	MASLD	180 (42.3)	130 (72.2)	50 (27.8)	
	Other	113 (26.5)	99 (87.6)	14 (12.4)	
Cirrhosis	Yes	188 (44.1)	113 (60.1)	55 (28.2)	0.03
Dyslipidaemia	Yes	122 (28.6)	98 (80.3)	24 (19.7)	0.169
T2DM	Yes	176 (41.3)	126 (71.6)	50 (28.4)	0.087
Hypertension	Yes	218 (51.2)	163 (74.8)	55 (25.2)	0.604
CVD	Yes	91 (21.4)	69 (75.8)	22 (24.2)	0.999
Thyroid disorders	Yes	48 (11.3)	39 (81.3)	9 (18.8)	0.351
Anxiety	Yes	82 (19.2)	57 (69.5)	25 (30.5)	0.138
Depression	Yes	112 (26.3)	76 (67.9)	36 (32.1)	0.022
Psoriasis	Yes	45 (10.6)	29 (64.4)	16 (35.6)	0.059
PCOS	Yes	5 (1.2)	3 (60.0)	2 (40.0)	0.598
		**Count (%)**	**Count (%)**	**Count (%)**	
**Retail outlet**	Lower cost	215 (44.6)	158 (73.5)	57 (26.5)	0.396
	Mid cost	216 (44.8)	164 (75.9)	52 (24.1)	
	Higher cost	51 (10.6)	34 (66.7)	17 (33.3)	
		**Mean ± SD**	**Mean ± SD**	**Mean ± SD**	
IOD Rank	—	87 ± 33.7	88 ± 34.4	85 ± 31.4	0.279*

*Note:* Tests for group differences performed with Chi Square or Fisher Freeman Hamilton (if cell count less than 5).

Abbreviations: ArLD, Alcohol related liver disease; CVD, Cardiovascular disease; IOD, Index of Deprivation; MASLD, Metabolic dysfunction associated liver disease; M&S, Marks and Spencer's; PCOS, Polycystic Ovarian Syndrome; T2DM, Type 2 Diabetes Mellitus.

* Mann‐Whitney U test for non‐parametric continuous data.

### 3.2 Associations of Food Insecurity With Clinical and Sociodemographic Variables

A total of 103 participants (24.2%) were identified as experiencing food insecurity based on their responses in our screening process: two or more affirmative indicators in the USDA six‐item component [14], and/or one or more affirmative response in the Hunger Vital Signs screening component [15]. Food insecurity prevalence amongst our sample was signficantly higher than the UK general population prevalence (14.8%, *p* = 0.021), based on data from 2024 [8, 21]. Of the food insecure group, 59 (57.3%) were seen by the dietitian for the brief intervention. The characteristics of this sub‐group are shown in Table 2. The reasons the brief intervention was not completed included patients declining the review or non‐referral. Non‐referrals were related to incorrect scoring, thus food insecurity was identified retrospectively.

**Table 2:** Sample socio‐demographic and clinical characteristics by food security severity: low vs very low food security.

Characteristics	Category	Total	Low food security	Very low food security	*p* value
		*n* = 59	*n* = 36 (61.0)	*n* = 23 (39.0)	
		*n* (%)	*n* (%)	*n* (%)	
		Mean ± SD	Mean ± SD	Mean ± SD	
Household occupants	Adult only households	39 (66.1)	24 (61.5)	15 (38.5)	
	Households with children ( < 18years)	20 (33.9)	12 (60.0)	8 (40.0)	
Age, years	18–24	2 (3.4)	2 (100.0)	0 (0.0)	0.87
	25–34	2 (3.4)	1 (50.0)	1 (50.0)	
	35–44	15 (25.4)	8 (53.3)	7 (46.7)	
	45–54	16 (27.1)	11 (68.8)	5 (31.3)	
	55–64	14 (23.7)	8 (57.1)	6 (42.9)	
	65 and over	10 (16.9)	7 (70.0)	3 (30.0)	
Gender	Male	35 (59.3)	19 (54.3)	16 (45.7)	0.106
Ethnicity	White British/other	55 (93.2)	35 (63.6)	20 (36.4)	0.718
	Black ‐ any black background	0 (0.0)	0 (0.0)	0 (0.0)	
	Asian ‐ any Asian Background	1 (1.7)	0 (0.0)	1 (100.0)	
	Other	3 (5.1)	2 (66.7)	1 (33.3)	
Marital status	Married/civil partner	16 (27.1)	12 (75.0)	4 (25.0)	0.122
	Single	13 (22.0)	5 (38.5)	8 (61.5)	
	Divorced/separated/widowed	3 (5.1)	1 (33.3)	2 (66.7)	
Smoking status	Smoker	9 (15.3)	5 (55.6)	4 (44.4)	0 931
	Ex‐smoker	23 (39.0)	14 (60.9)	9 (39.1)	
	Nonsmoker	22 (37.3)	15 (68.2)	7 (31.8)	
Alcohol intake (Audit‐C)	0–4 (low risk)	50 (8.5)	32 (64.0)	18 (36.0)	0.37
	5–7 (increasing risk)	1 (4.5)	0 (0.0)	1 (100.0)	
	8–12 (high risk/dependence)	6 (10.2)	3 (50.0)	3 (50.0)	
BMI	18.5–24.9	8 (13.6)	4 (50.0)	4 (50.0)	0.568
	25–29.9	10 (16.9)	5 (50.0)	5 (50.0)	
	30–34.9	17 (28.8)	12 (70.6)	5 (29.4)	
	35–39.9	14 (23.7)	10 (71.4)	4 (28.6)	
	40–49.9	7 (11.9)	4 (57.1)	3 (42.9)	
	>/= 50	0 (0.0)	0 (0.0)	0 (0.0)	
HbA1c	< 42	26 (44.1)	18 (69.2)	8 (30.8)	0.21
	42–47	15 (25.4)	9 (60.0)	6 (40.0)	
	48–57	2 (3.4)	2 (100.0)	0 (0.0)	
	>/= 58	16 (27.1)	8 (50.0)	8 (50.0)	
Diagnosis	ArLD	15 (25.4)	8 (53.3)	7 (46.7)	0.304
	Viral hepatitis	5 (8.5)	2 (40.0)	3 (60.0)	
	MASLD	31 (52.5)	20 (64.5)	11 (35.4)	
	Other	8 (13.6)	7 (87.5)	1 (12.5)	
Cirrhosis	Yes	31 (52.5)	21 (67.7)	10 (32.3)	0.401
Dyslipidaemia	Yes	14 (23.7)	7 (50.0)	7 (50.0)	0.345
T2DM	Yes	30 (50.8)	16 (53.3)	14 (46.7)	0.13
Hypertension	Yes	32 (54.2)	21 (65.6)	11 (34.4)	0.614
Cardiovascular disease	Yes	13 (22.0)	7 (53.8)	6 (46.2)	0.524
Thyroid disorders	Yes	7 (11.9)	3 (42.9)	4 (57.1)	0.407
Anxiety	Yes	16 (27.1)	10 (62.5)	6 (37.5)	0.984
Depression	Yes	23 (39.0)	16 (69.6)	7 (30.4)	0.384
Psoriasis	Yes	12 (20.3)	6 (50.0)	6 (50.0)	0.334
PCOS	Yes	2 (5.4)	2 (100.0)	0 (0.0)	0.524

*Note:* Tests for group differences performed with Chi Square or Fisher Freeman Hamilton (if cell count less than 5).

Abbreviations: ArLD, Alcohol related liver disease; CVD, Cardiovascular disease; IOD, Index of Deprivation; MASLD, Metabolic dysfunction associated liver disease; PCOS, Polycystic Ovarian Syndrome; T2DM, Type 2 Diabetes Mellitus. *Mann‐Whitney U test for non‐parametric continuous data.

Food insecurity prevalence varied by age (Fisher‐Freeman‐Halton Exact Test: 27.3 p =< 0.001), marital status (Pearson Chi‐square: 7.2, *p* = 0.028), alcohol intake (Fisher‐Freeman‐Halton Exact Test: 12.3, *p* = 0.006), diagnosis (Pearson Chi‐square: 14.5, *p* = 0.002), presence of cirrhosis (Pearson Chi‐square: 4.7, *p* = 0.03) or depression (Pearson Chi‐square: 5.3, *p* = 0.022). There were no significant differences between food secure and food insecure groups (table 1) relating to gender, Index of Multiple Deprivation (IOD) rank, smoking status, BMI, Hba1c, dyslipidaemia, T2DM, hypertension, cardiovascular disease, thyroid disorders, anxiety, psoriasis, PCOS or primary retail outlet. No statistically significant differences were observed between any groups when comparing very low (*n *= 23; 39%) with low food security (*n *= 36; 61%) (table 2). However, only 57% of food insecure participants (*n* = 59) completed extended screening related to food insecurity severity and therefore this is not representative of the total sample.

In binary logistic regression (table 3), predictive variables for food insecurity were identified as: age range 35‐44 years (odds ratio = 2.915, 95% CI: 1.599–5.313, *p* = < 0.001), age 45–54 years (odds ratio = 2.278, 95% CI: 1.290–4.023, *p* = 0.005), high risk or possible dependent alcohol intake (odds ratio = 2.715, 95% CI: 1.091–6.756, *p* = 0.032), alcohol related liver disease aetiology (odds ratio = 1.860, 95% CI: 1.102–3.138, *p* = 0.02), cirrhosis (odds ratio = 1.637, 95% CI: 1.051–2.560, *p* = 0.03) and depression (odds ratio = 1.746, 95% CI: 1.081–2.821, *p* = 0.023). Comparatively, food insecurity was found to be significantly less likely in age range over 65 years (odds ratio = 0.412, 95% CI: 0.248–0.685, *p* = < 0.001) and marriage/civil partnership (odds ratio = 0.607, 95% CI: 0.377–0.977, *p* = 0.04).

**Table 3:** Logistic regression showing associations between food insecurity and study variables.

Characteristics	Category	OR (95% CI)	*p* value
Age, years	18–24	1.3 (0.2–7.0)	0.785
	25–34	1.4 (0.5–3.6)	0.535
	35–44	2.9 (1.6–5.3)	< 0.001
	45–54	2.3 (1.3–4.0)	0.005
	55–64	0.7 (0.4–1.1)	0.138
	65 and over	0.4 (0.2–0.7)	< 0.001
Gender	Male	1.2 (0.7–1.8)	0.539
Ethnicity	White British/other	1.1 (0.4–2.7)	0.892
	Black and Asian	6.2 (0.6–69.6)	0.136
	Other	0.5 (0.1–4.4)	0.548
Marital status	Married/civil partner	0.6 (0.4–1.0)	0.04
	Single	0.9 (0.5–1.6)	0.823
	Divorced/separated/widowed	1.5 (0.5–4.0)	0.443
IOD Rank	‐‐‐	1.0 (1.0–1004)	0.429
Smoking status	Smoker	1.4 (0.7–2.7)	0.308
	Ex‐smoker	1.1 (0.7–1.7)	0.792
	Nonsmoker	0.7 (0.4–1.1)	0.144
Alcohol intake (Audit‐C)	0–4 (low risk)	1.0 (0.5–1.9)	0.887
	5–7 (increasing risk)	0.4 (0.1–1.2)	0.117
	8–12 (high risk/dependence)	2.7 (1.1–6.8)	0.032
BMI	18.5–24.9	1.0 (0.5–1.7)	0.886
	25–29.9	0.7 (0.4–1.2)	0.236
	30–34.9	1.1 (0.7–1.8)	0.733
	35–39.9	1.3 (0.8–2.2)	0.319
	40–49.9	1.2 (0.6–2.6)	0.609
	>/= 50	0.5 (0.6–5.0)	0.564
HbA1c	< 42	0.8 (0.5–1.3)	0.347
	42–47	1.2 (0.7–2.0)	0.415
	48–57	0.4 (0.1–1.2)	0.098
	>/= 58	1.7 (1.0–2.9)	0.062
Diagnosis	ArLD	1.9 (1.1–3.1)	0.02
	Viral hepatitis	1.0 (0.5–2.0)	0.965
	MASLD	1.4 (0.9–2.2)	0.139
	Other	0.3 (0.2–0.7)	< 0.001
Cirrhosis	Yes	1.6 (1.1–2.6)	0.03
Dyslipidaemia	Yes	1.4 (0.9–2.4)	0.17
T2DM	Yes	0.7 (0.4–1.1)	0.088
Hypertension	Yes	0.9 (0.6–1.4)	0.604
Cardiovascular disease	Yes	1.0 (0.6–1.7)	0.999
Thyroid disorders	Yes	1.4 (0.7–3.1)	0.353
Anxiety	Yes	0.7 (0.4–1.1)	0.139
Depression	Yes	1.7 (1.1–2.8)	0.023
Psoriasis	Yes	0.5 (0.3–1.0)	0.063
PCOS	Yes	0.5 (0.1–2.9)	0.416

Abbreviations: ArLD, Alcohol related liver disease; CVD, Cardiovascular disease; IOD, Index of Deprivation; MASLD, Metabolic dysfunction associated liver disease; PCOS, Polycystic Ovarian Syndrome; T2DM, Type 2 Diabetes Mellitus.

### 3.3 Associations of Food Insecurity With Liver Related Morbidity

Logistic regression models (table 4) showed food insecurity to be associated with ArLD compared to other diagnoses without adjustment (AOR = 1.9, 95%CI 1.1–3.1, *p* = 0.02). When adjusted for age, food insecurity was associated with both ArLD (AOR = 4.4, 95%CI 2.0–9.6, *p* = < 0.001) and MASLD (AOR = 3.4, 95%CI 1.7–6.8, *p* = < 0.001) compared to other diagnoses. This association was weakened after adjustment for both sociodemographic variables and co‐morbidities in model 3 (Adjusted for Age, marital status, alcohol intake, depression) (ArLD: AOR = 3.2, 95%CI 1.3–8.0, *p* = 0.013; MASLD: AOR = 2.3, 95%CI 1.1–5.0, *p* = 0.03). Food insecurity was significantly associated with cirrhosis in the unadjusted model (AOR = 1.6, 95%CI 1.1–2.6, *p* = 0.03), with a stronger association after adjustment for age and diagnosis (model 2) (AOR = 1.9, 95%CI 1.1–3.2, *p* = 0.024), and clinico‐sociodemographic characteristics (model 3) (AOR = 1.9, 95% CI 1.0–3.0, *p* = 0.034) (table 5).

**Table 4:** The Relationship between food insecurity and diagnosis based on logistic regression test.

			AOR (95% CI)	*p* value
Model 1	Diagnosis	ArLD	1.9 (1.1–3.1)	0.02
		Viral hepatitis	1.0 (0.5–2.0)	0.965
		MASLD	1.4 (0.9–2.2)	0.139
		Other	Ref	
Model 2	Diagnosis	ArLD	4.4 (2.0–9.6)	< 0.001
		Viral hepatitis	1.5 (0.6–3.7)	0.39
		MASLD	3.4 (1.7–6.8)	< 0.001
		Other	Ref	
Model 3	Diagnosis	ArLD	3.2 (1.3–8.0)	0.013
		Viral hepatitis	2.1 (0.8–5.6)	0.152
		MASLD	2.3 (1.1–5.0)	0.03
		Other	Ref	

*Note:* Model 1 – Unadjusted; Model 2 – Adjusted for age; Model 3 ‐ Adjusted for Age, marital status, alcohol intake, depression.

Abbreviations: ArLD, Alcohol related liver disease; MASLD, Metabolic dysfunction associated liver disease.

**Table 5:** ‐ The Relationship between food insecurity and cirrhosis based on logistic regression test.

	Category	OR (95% CI)	*p* value
Model 1	Cirrhosis	1.6 (1.1–2.6)	0.03
Model 2	Cirrhosis	1.9 (1.1–3.2)	0.024
Model 3	Cirrhosis	1.9 (1.0–3.0)	0.034

*Note:* Model 1 – Unadjusted; Model 2 – Adjusted for age and diagnosis; Model 3 ‐ Adjusted for Diagnosis, Age, Marital status, alcohol intake, depression.

## 4 Discussion

This cross‐sectional study aimed to evaluate the prevalence of food insecurity and its associated clinico‐sociodemographic factors in patients attending hepatology outpatient clinics. Our findings show food insecurity to be highly prevalent amongst patients with liver disease. Associations were observed between food insecurity, alcohol‐related and metabolic dysfunction associated disease aetiologies, and more advanced disease (cirrhosis). Our data confirms existing evidence linking food insecurity with MASLD [22, 23]. Further, it extends on prior studies that demonstrated associations with more advanced disease in the form of advanced fibrosis [24, 25].

The present study demonstrated food insecurity prevalence of 24.2% amongst patients with chronic liver disease, higher than the UK general population prevalence of 14.8% [8, 26]. This is consistent with evidence from the USA, which identified associations between food insecurity and liver disease in large cohort cross‐sectional analyses [24, 27, 28]. However, there is limited data from UK based studies and current evidence focuses predominantly on MASLD rather than all‐cause aetiology. This elevated prevalence highlights a need to consider implications in development and management of liver disease, to attenuate existing health inequities.

In the current study food insecurity was significantly associated with ArLD and higher risk alcohol intake or alcohol dependence both without and after adjustment for socio‐demographic and clinical variables. There is a scarcity of studies that have explored the associations between food insecurity and ArLD. However, data from the USA evidenced a 23% higher risk of problematic alcohol use amongst food insecure individuals [29]. Importantly, both food insecurity and harmful alcohol consumption have increased since the COVID‐19 pandemic [8, 30, 31] with these increases disproportionately impacting the economically disadvantaged [30]. Similarly, alcohol related hospital admissions and mortality have risen [31]. Whilst associations between harmful alcohol intake, ArLD and food insecurity have been evidenced in this study and existing literature, the nature of this relationship remains undefined. Future research should address this to facilitate directed support, particularly in the context of parallel rising burdens in food insecurity and ArLD.

The prevalence of MASLD was higher in the food insecure group (48.5% vs 40.5%), and food insecurity was significantly associated with greater odds of MASLD after adjustment for both socio‐demographic and clinical‐sociodemographic variables. Recent studies suggest food insecurity as a risk factor for MASLD [22, 23, 32] and have identified associations between food insecurity, mortality and healthcare utilisation amongst MASLD patients [24]. Dietary modifications, particularly the Mediterranean Diet, are a cornerstone of MASLD prevention and management [3] and adherence is reduced amongst those with lower food related expenditure [33]. There is evidence that dietary substitutions are made by food insecure populations towards cheaper, energy dense foods often higher in sugar and saturated fats [11, 34], which are associated with MASLD development [34]. Cyclical eating patterns driven by fluctuations in food availability may further disrupt metabolism amongst food insecure individuals [35]. Food insecurity contributes to multifactorial risks for MASLD development and progression, whilst additionally presenting an important barrier to uptake of dietary interventions for MASLD management. It should therefore be routinely considered when working with this patient group. Further, there is need for targeted interventions that address the specific needs and complexities of this population.

Recent evidence has indicated a relationship between food insecurity and advanced fibrosis [18, 19]. This is in accordance with the results of the present study, in which food insecurity was associated with increased odds of cirrhosis. The causal factors underpinning these associations warrant exploration but were outside the scope of this study.

Our data suggests an association between depression and food insecurity, reflecting the bidirectional relationships which have been previously evidenced elsewhere [36, 37]. This is notable amongst our sample as risk factors for liver disease: excess alcohol intake, reduced diet quality and obesity are closely linked with depression [38, 39]. Food insecure households report increased feelings of isolation, guilt and shame, which may impact their engagement with services [40]. The stigma associated with food insecurity may be a contributing factor in its associations with adverse mental health, and potentially exacerbated amongst those living with liver disease, which is known to be a stigmatised condition [41, 42]. Therefore, it is important that both food insecurity and psychological screening and support is sensitively incorporated into practice.

Concerning sociodemographic characteristics, food insecurity was more prevalent in those aged 35–44 years (22.3% vs 9.0% food secure) and 45–54 years (23.3% vs 11.8% food secure). Food insecurity prevalence was lower amongst those who were married or in a civil partnership (18.4% vs 32.1% food secure). These findings reflect general population data demonstrating higher rates of food insecurity amongst working age, single adults [21].

This study highlights limitations to food insecurity screening within the clinical setting, reflected by a 33% nonresponse rate. Of the study participants who were identified as food insecure, only 57% received a brief intervention with the dietitian. Seemingly, there are challenges to food insecurity screening and the provision of related support in the clinical setting. It is feasible that these arise from a combination of patient‐related factors such as perceived stigma and language barriers [9], alongside well documented barriers to change within healthcare systems [43]. Future research is needed to examine these barriers through qualitative and implementation‐focused approaches, involving both clinical staff and patients. This should aim to develop robustly integrated, inclusive and sustainable screening and referral pathways.

Limited evidence on current interventions for food insecurity in clinical practice outside hepatology suggest a focus on education, particularly budgeting and cooking skills [44]. However, population data shows that food insecure households have similar food‐related knowledge and skills to food secure households [45]. The efficacy of interventions targeting food preparation and planning is therefore likely to be limited. Support may also take the form of signposting or referral to food banks or similar community food access initiatives [44], but the effectiveness and acceptability of these interventions among the general population are limited. Moreover, there is evidence to suggest that the food provision from food banks are disproportionately high in sugar and carbohydrates, and lacking key micronutrients [46]. Thus, this may negatively impact the dietary quality of people accessing these facilities and worsen health outcomes. Furthermore, it may reduce individuals' adoption of dietary recommendations in liver disease. More data is needed from research utilising codesign methods with clinicians and patients to inform the development of effective, feasible and acceptable interventions for this population group.

The primary driver of food insecurity is economic. Specifically, income constraints combined with rising food costs and household expenses underpin the rising prevalence of food insecurity amongst the UK general population [7]. Several studies demonstrate that strategies used by food insecure individuals, such as budgeting, meal planning and seeking additional income, are minimally effective in improving their food security status [47]. Clinical and community interventions to address food insecurity may offer only partial support for those affected. For sustainable change in food insecurity prevalence and impact, the root economic causes must be addressed.

The main strengths of the current study include the use of validated screening measures for food insecurity, and a sample which includes diversity across liver diagnoses and patient characteristics. The data collection period was of sufficient duration to minimise the impact of short‐term, seasonal fluctuations in patients perceived food security status which may have biased their responses. Further, patients were offered brief face to face interventions with dietitians where signposting and support was provided. The completion of food insecurity screening before face‐to‐face consultations also gave opportunity to address any difficulties in completion of the screening tool, such as where literacy presented a barrier. This enabled some patients to complete screening where it may not have been feasible via methods such as online reports.

Limitations of this study include its cross‐sectional design which limits the consideration of causal relationships. There is a risk of participation bias arising from the voluntary recruitment method. Further, whilst comparisons have been made to the UK general population based on existing evidence, a lack of comparative group in the current study prevents direct comparison to the regional population or timeframe studied. Our sample comprised a high prevalence of white British participants (*n* = 400, 93.9%), which may impact findings. A smaller number of patients completed extended screening, which impacts the interpretation of the data concerning severity of food insecurity across all variables. Self‐reported measures of food security status, such as those used in this study, have been associated with under‐reporting of food insecurity [48]. Further, translated versions of the screening tools may have improved accuracy of completion across different languages. However, these tools have been demonstrated to offer diagnostic accuracy, are accessible and time‐efficient particularly within the constraints of clinical practice [49].

Future research should aim to include a larger and more diverse sample from a wider geographical location when considering food insecurity severity. Further, the inclusion of a comparative group and capturing data on disabilities, household income, household occupants, employment status and fibrosis stage would be beneficial to enhance our understanding of food insecurity relationships in this disease population.

## 5 Conclusion

This study found that food insecurity was highly prevalent amongst patients with chronic liver disease in Northern England, and was associated with ArLD, MASLD and cirrhosis. Previous studies have highlighted that food insecurity is associated with reduced diet quality, impaired nutritional status and obesity. Thus, it presents an obstacle to dietary modifications which are often recommended across a spectrum of liver disorders. Healthcare professionals working in hepatology services should consider routinely screening for food insecurity and provide appropriate support to mitigate this health inequality, particularly in patients living with cirrhosis. Further research is needed to determine intervention approaches, which can effectively identify and address food insecurity in clinical practice. These studies should include codesign strategies between services, clinicians and patients to optimise effectiveness, feasibility and acceptability.

## Author Contributions

L.H., Q.M.A., S.M., R.H., T.C. formulated the research questions and developed the study protocol. L.H., Q.M.A., S.M., R.H., T.C. collected the data, with R.H. extracting the data and L.H., T.C. providing supervision. R.H. analysed the data with supervision from statistical analysis staff at Newcastle University. R.H. wrote the manuscript. L.H., Q.M.A., S.M., and T.C. reviewed the manuscript, and all authors had final responsibility for the decision to submit for publication.

## Conflicts of Interest

The authors declare no conflicts of interest.
